# Intermittent Hypoxia Composite Abnormal Glucose Metabolism-Mediated Atherosclerosis *In Vitro* and *In Vivo*: The Role of SREBP-1

**DOI:** 10.1155/2019/4862760

**Published:** 2019-02-04

**Authors:** Linqin Ma, Jingchun Zhang, Yu Qiao, Xinli Sun, Ting Mao, Shuyan Lei, Qiaoxian Zheng, Yue Liu

**Affiliations:** ^1^Cardiovascular Diseases Center, Xiyuan Hospital, China Academy of Chinese Medical Sciences, Beijing 100091, China; ^2^Department of Emergency, Dongzhimen Hospital, Beijing University of Chinese Medicine, Beijing 100700, China; ^3^Cardiopulmonary Division, TCM Hospital of Beijing Mentougou District, Beijing 102300, China; ^4^Graduate School, Beijing University of Chinese Medicine, Beijing 100029, China

## Abstract

**Objective:**

The aim of this study was to establish a 3T3-L1 adipocyte model and ApoE^−/−^ mouse model of intermittent hypoxia (IH) composite abnormal glucose metabolism (AGM) *in vitro* and *in vivo* and explore their synergistic damage effect leading to atherosclerosis (AS) and the influence of SREBP-1 signaling molecule-related mechanisms.

**Methods:**

Mature 3T3-L1 adipocytes were cultured with complete culture medium containing DEX 1 × 10^6^ mol/L for 96 h to establish an AGM-3T3-L1 adipocyte model. Then, AGM-3T3-L1 adipocytes were treated with IH for 0 cycles, 2 cycles, 4 cycles, 8 cycles, 16 cycles, and 32 cycles and sustained hypoxia (SH). ApoE^−/−^ mice were treated with high-fat diet and injection of STZ solution to establish an AGM-ApoE^−/−^ mouse model. A total of 16 AGM-ApoE^−/−^ mice were randomly and averagely divided into the normoxic control group (NC) and model group (CIH). AGM-ApoE^−/−^ mice of the CIH group were treated with IH, which meant that the oxygen concentration fell to 10 ± 0.5% in the first 90 seconds of one cycle and then increased to 21 ± 0.5% in the later 90 seconds, continuous for eight hours per day (09 : 00-17 : 00) with a total of eight weeks. Eight C57BL/6J mice were used as the blank control group (Con) which was fed with conventional diet. qPCR and Western blotting were used to detect the expression level of SREBP-1c, FAS, and IRS-1. Oil Red O staining was used to compare the plaque of the aorta among each mouse group.

**Results:**

As a result, within 32 cycles of IH, mRNA and protein expression levels of SREBP-1c and FAS in AGM-3T3-L1 adipocytes were elevated with the increase in IH cycles; the mRNA expression of IRS-1 was decreased after IH 32 cycles and lower than that of the SH group. For the study *in vivo*, Oil Red O staining showed a more obvious AS aortic plaque in the CIH group. After CIH treatment of 4 w and 8 w, fasting blood glucose (FBG) of the NC group and CIH group was higher than that of the Con group, and the insulin level of the CIH group was higher than that of the Con group after IH treatment of 8 w. The expressions of the IRS-1 mRNA level in the aorta, skeletal muscle, and liver of the CIH group were lower than those in the Con group. The mRNA and protein expression of SREBP-1c and its downstream molecule FAS in the aorta, skeletal muscle, and liver significantly enhanced in the CIH group in contrast with those in the Con group.

**Conclusion:**

The CIH composite AGM could promote the progress of AS, which might be related to the modulation of the expression of SREBP-1-related molecular pathways.

## 1. Introduction

Obstructive sleep apnea-hypopnea syndrome (OSAHS), which has high morbidity, is an independent risk factor for cardiovascular diseases and that predisposes to hypertension, myocardial infarction, heart failure, and even sudden cardiac death during the nighttime. Chronic intermittent hypoxia (CIH), which is a pivotal physiopathological character of OSAHS, could lead to the deterioration of the insulin resistance and the formation of glucose and lipid metabolic disorder. The synergy of CIH and abnormal glucose metabolism (AGM) can promote vascular endothelial injury and cause OSAHS-related atherosclerosis (AS) and other vascular lesions. By reviewing and summarizing current researches, we found that some experimental researches have preliminarily studied the possible mechanisms related to intermittent hypoxia (IH), insulin resistance, or AGM and AS. For example, literature had suggested the following possible signaling molecules or pathways: hypoxia exposure regulated the expression of lysyl oxidase and other target genes via promotion of HIF-1; the HIF-1/Angptl4 pathway may play a vital role in CIH-mediated abnormal lipid metabolism and even atherosclerosis formation; and SREBPs, which are closely related to glycolipid metabolism, might be related to CIH-mediated insulin resistance. It could also be seen that there are more concerns about the relationship between IH and AGM or IH and AS, while it was rare to consider IH and AGM as costimulatory factors in mediating AS. In clinical practice, as we know, AGM, dyslipidemia, obesity, and other insulin resistance-based metabolic disorders often occur with OSAHS simultaneously, and the synergistic effect of IH and AGM on AS might be greater than the simple sum of the effect of each one. This was the reason why it was necessary to observe the synergistic effect of CIH and AGM-mediated AS under experimental conditions to initially answer the above questions provided by insufficient information. This study built a model system of CIH composite AGM, both *in vitro* and *in vivo*, to observe SREBP-1 signaling molecule-related mechanisms.

## 2. Materials and Methods

### 2.1. Materials

The embryo fibroblasts of 3T3-L1 were obtained from the American Type Culture Collection (ATCC). High-glucose Dulbecco's modified eagle medium (H-DMEM, HyClone, SH30022), fetal bovine serum (FBS, Gibco, 10099141), phosphate-buffered saline (PBS, HyClone, SH30256), NuPAGE MOPS SDS Running Buffer (NP0001), iBlot Transfer Stack (IB301001), PageRuler Prestained Protein Ladder (26617), SuperSignal West Femto Maximum Sensitivity Substrate (34905), and TRIzol Reagent (Invitrogen, 15596-026) were purchased from Thermo Fisher Scientific. 3-Isobutyl-1-methylxanthine (IBMX, I5879), insulin (I5500), dexamethasone (DEX, D1756), glucose assay kit (GAGO20), and diethyl pyrocarbonate-treated water (DEPC, 95284) were purchased from Sigma-Aldrich. PrimeScript RT Master Mix (RR036A) and SYBR Premix Ex Taq II (RR420A) were purchased from TaKaRa. SREBP-1 antibody (ab28481), anti-fatty acid synthase (FAS) antibody (ab22759), and p-IRS-1 antibody (ab5599) were purchased from Abcam. *β*-Actin antibody (TA-09), GAPDH (TA-08), and other secondary antibodies were purchased from ZSGB-BIO. The Modular Incubator Chamber (MIC-101) was purchased from Billups-Rothenberg Inc. The hypoxic animal incubator (YCP-160D) was purchased from Changsha Huaxiao Electronic Technology Co. Ltd. Other laboratory equipment included Real-Time Quantitative PCR detecting system (ABI 7500, Life Tech) and Western blot electrophoresis (Thermo Fisher Scientific). Primer sequences are as follows: *β*-actin mouse F-CCATGTACGTAGCCATCCAG, *β*-actin mouse R-GAGTCCATCACAATGCCTGT, SREBP-1c mouse F-TTGTGGAGCTCAAAGACCTG, SREBP-1c mouse R–TGCAAGAAGCGGATGTAGTC, IRS-1 mouse F–ATTAAACACTGGGCCTCTGG, and IRS-1 mouse R–GCTGTGCCATACTCTCTCCA (synthesized by Sangon Biotech, Shanghai).

### 2.2. Animals

Male 8-week-old ApoE^−/−^mice and 8-week-old C57BL/6J mice were purchased from Beijing Vital River Laboratory Animal Technology Co. Ltd. The weight of mice was 23 ± 1 g. All animals were fed in the Animal Experimental Center of Xiyuan Hospital of China Academy of Chinese Medical Sciences with room temperature of 22 ± 2°C, relative humidity of 55 ± 5%, and light time 07:00-19:00. After adaptive feeding for a week, the experimental phase began. During the experimental period, all ApoE^−/−^ mice were fed with high-fat feed. The composition of a high-fat diet [1, 2] consisting of 21% fat, 0.15% cholesterol, and 78.85% conventional basic feed, was purchased from Beijing Hua Fukang Biotechnology Co. Ltd. C57BL/6J mice were given conventional basic feed. Every 4 weeks, the weight of the mice and fasting blood glucose would be detected.

### 2.3. Cell Culture and Differentiation

**Figure 1:**
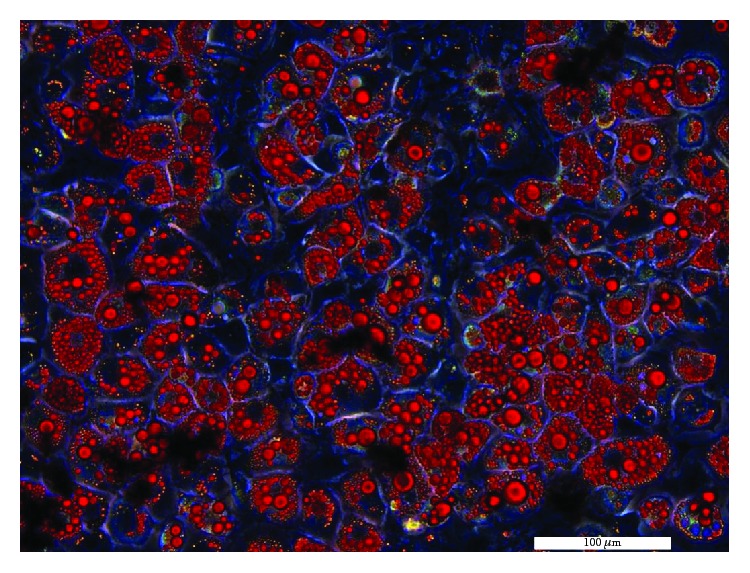
Differentiated and mature 3T3-L1 adipocytes (Oil Red O staining).

Conventional culture, inheritance, cryopreservation, and anabiosis of the embryo fibroblasts of 3T3-L1 had been done routinely. Two days after contact inhibition of the embryo fibroblasts, for differentiation, the cell culture medium would be replaced by cell differentiation solution I, of which the composition contained 0.5 mmol/L IBMX, 5 *μ*g/mL insulin, 1 *μ*mol/L DEX, and complete cell culture medium. After 2 days, the medium would be changed into cell differentiation solution II, of which the composition contained 5 *μ*g/mL insulin and complete cell culture medium, after culture with a cell differentiation solution II for 8-11 days. For verification by the method of Oil Red O staining, the rate of cell differentiation could reach more than 80% and 3T3-L1 preadipocytes were successfully differentiated into mature 3T3-L1 adipocytes (Figure 1).

### 2.4. Establishment of Cell Model of IH Composite AGM

Mature 3T3-L1 adipocytes were treated with a complete medium containing DEX 1 *μ*mol/L for 96 h to establish a cell model of AGM. The method of glucose oxidase-peroxidase (GOD-POD) was used to detect the glucose concentration in the cell culture medium. The result showed that the glucose concentration in the group pretreated with the cell culture medium with DEX after 96 h was higher than that of the control group (*n* = 12, *P* < 0.05). These AGM-3T3-L1 adipocytes would be treated with IH to establish the cell model of IH composite AGMT3-L1 adipocytes.

**Figure 2:**
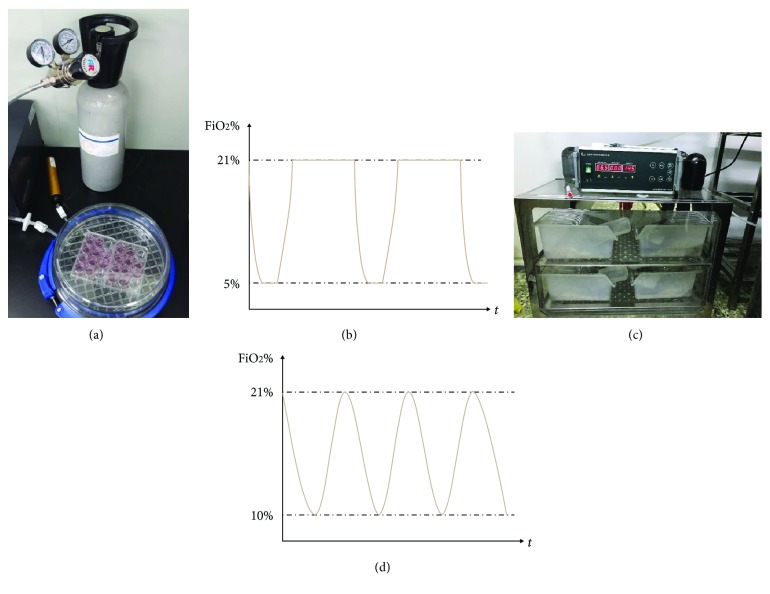
Schematic diagram of IH treatment (*in vitro*) and CIH treatment (*in vivo*). (a) Schematic diagram of IH treatment (*in vitro*); (b) IH treatment cycle pattern (*in vitro*); (c) schematic diagram of CIH treatment (*in vivo*); (d) CIH treatment hypoxia cycle pattern (*in vivo*).

The specific procedure was as follows: (1) AGM-3T3-L1 adipocytes were replaced with H-DMEM without FBS and cultured for 0.5 h in a cell incubator at 37°C. (2) Hypoxia device MIC-101 was UV-disinfected. (3) Cell culture dishes were placed in MIC-101, while the oxygen electrode was used to monitor the oxygen concentration at the gas outlet. During the hypoxia phase, the air in the chamber of MIC-101 was eluted with low oxygen air containing 1% O_2_, 5% CO_2_, and 94% N_2_. When the oxygen concentration reaches 5%, the intake of low oxygen air would be stopped and the inlet and outlet would be closed. This hypoxia condition would be maintained for 1 minute. Then, the reoxygen phase was started, and oxygen was passed into the chamber. When the oxygen concentration at the outlet reaches 21%, the intake of oxygen would be stopped and the inlet and outlet would be closed. This normal oxygen condition would be maintained for 5 minutes. The hypoxia and reoxygen cycle would be repeated for 2, 4, 8, 16, and 32 cycles for different groups of IH (IH2, IH4, IH8, IH16, and IH32) (Figure 2). (4) Another cell culture dish of AGM-3T3-L1 adipocytes would be treated by sustained hypoxia (SH) at the same time, which meant the cells were cultured in an oxygen incubator at an oxygen concentration of about 5% for about 4 hours, which is the same as in the IH32 group.

### 2.5. Establishment of Animal Model of CIH Composite AGM

The AGM-ApoE^−/−^ mouse model was established by high-fat feed and streptozocin (STZ) injection. 16 ApoE^−/−^ mice, as the AGB group, were given a high-fat diet for 4 weeks, and then intraperitoneally injected with STZ solution at a dose of 50 mg/kg for 5 consecutive days [3]. AGM-ApoE^−/−^ mice were randomly divided into 2 groups. Eight C57BL/6J mice, as the control group, were injected with the same volume of sterilized injection water at the same time. After 72 h, fasting blood glucose (FBG) of each group was detected. The blood glucose in the AGB group (9.31 ± 1.32 mmol/L) was significantly higher than that in the control group (6.05 ± 0.43 mmol/L) (*P* < 0.05), which meant mice in the AGB group could be used for the next experiment.

These AGB-ApoE^−/−^ mice were randomly divided into normoxia control group (NC) and chronic intermittent hypoxia group (CIH) and were fed with high-fat diet. At the same time, eight C57BL/6J mice of the same week of age, as a blank control group (Con), were fed with conventional basic feed. Mice of the CIH group had been treated in a sealed incubator of hypoxia animal feeding device YCP-160D. Specific operational procedures are as follows: (1) put the mice of the CIH group into the incubator and confirm that the incubator door is closed tightly. (2) Switch on the power of YCP-160D and enter “intermittent hypoxia control” mode. (3) Switch on the gas pressure reducer of the N_2_ and O_2_ bottle to start ventilation. (4) In the “N_2_ intake” mode, adjust the gas pressure reducer of N_2_ so that the N_2_ outlet pressure is about 0.08 kPa, while in “O2 intake” mode adjust the gas pressure reducer of O_2_ so that the O_2_ outlet pressure is about 0.015 kPa. (5) Observe several CIH cycles to ensure that FIO_2_ of the incubator decreases to 10 ± 0.5% within the former 90 seconds of one cycle and increases to 21 ± 0.5% within the later 90 seconds of one cycle; the intake pressure was fine-tuned so that the highest and lowest FIO_2_ is relatively stable in each cycle. (6) CIH treatment lasts for 8 hours daily (09:00-17:00) with a total of 8 weeks (Figure 2).

### 2.6. Methods of Experimental Indicator Test

In the part of cell experiment, qPCR was used to detect the mRNA expression level of SREBP-1c, FAS, and IRS-1. Western blotting was used to detect the protein expression level of SREBP-1 and FAS. In the part of animal experiment, the serum insulin level was measured by ELISA assay at the end of the experiment. Oil Red O staining was used to compare the plaque of the aorta among each group. qPCR was used to detect the mRNA expression levels of SREBP-1c, FAS, and IRS-1 of the aorta, skeletal muscle, and liver, while Western blotting was used to detect SREBP-1c and FAS protein p-IRS-1 expression levels of the aorta, skeletal muscle, and liver.

### 2.7. Statistical Analysis

All measurement data were expressed as mean ± standard error ($\bar{x}\pm s$). Differences between different groups were analyzed by Statistical Package for the Social Sciences version 20.0 (SPSS 20.0). The *T*-test was used to compare the two groups, while one-way ANOVA was used to compare the multiple groups. For data that did not meet the normal distribution, the nonparametric Kruskal-Wallis *H* test was used to compare the groups' differences between levels. A value of *P* < 0.05 was considered as statistically significant.

## 3. Results

### 3.1. Effect of Different IH Cycles on AGM-3T3-L1 Adipocytes

**Figure 3:**
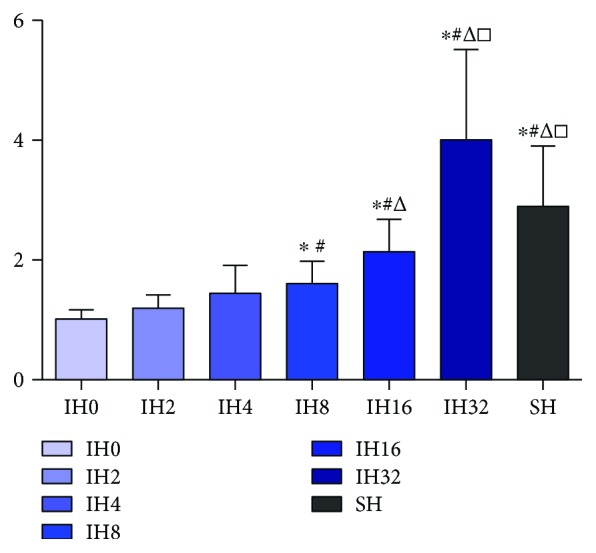
Effect of different numbers of IH cycles on SREBP-1c mRNA expression (*n* = 9) (compared with IH0 ^∗^*P* < 0.05, compared with IH2 ^#^*P* < 0.05, compared with IH4 ^△^*P* < 0.05, and compared with IH8 ^□^*P* < 0.05.)

**Figure 4:**
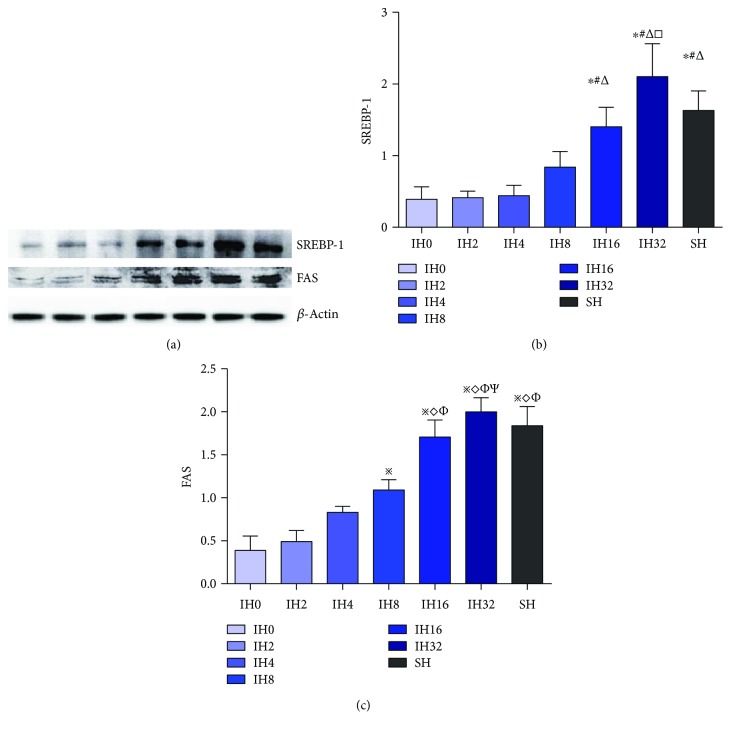
Effect of different numbers of IH cycles on SREBP-1 and FAS expression (*n* = 9) (a) Representative Western blotting result. (b) Compared with IH0 ^∗^*P* < 0.05, compared with IH2 ^#^*P* < 0.05, compared with IH4 ^△^*P* < 0.05, compared with IH8 ^□^*P* < 0.05. (c) Compared with IH0 ^※^*P* < 0.05, compared with IH2 ^◇^*P* < 0.05, compared with IH4 ^Φ^*P* < 0.05, and compared with IH8 ^Ψ^*P* < 0.05.

The expression level of SREBP-1c was focused on our experiment. The results of qPCR is shown in Figure 3. The expression level of SREBP-1c mRNA was elevated with the increase in the number of IH cycles within 32 cycles of IH treatment. Among these groups, the IH8, IH16, and IH32 groups and the SH group were statistically different with the IH0 group (*P* < 0.05); moreover, the IH32 group and SH group were statistically different with the IH8 group (*P* < 0.05). Results of Western blotting are shown in Figures 4(a) and 4(b). After 8 cycles of IH treatment, the expression level of the SREBP-1 protein was elevated with the increase in the number of IH cycles. Among them, the IH16 group, IH32 group, and SH group were statistically different from the IH0 group, IH2 group, and IH4 group (*P* < 0.05), and the IH32 group was significantly different from the IH8 group (*P* < 0.05).

Furthermore, we detected the protein expression level of FAS which is a downstream target molecule of SREBP-1c. Results are shown in Figures 4(a) and 4(c). Within 32 cycles of IH treatment, the expression level of the FAS protein increased with the number of IH, and the levels of the IH8 group, IH16 group, IH32 group, and SH group were statistically different from that of the IH0 group (*P* < 0.05).

**Figure 5:**
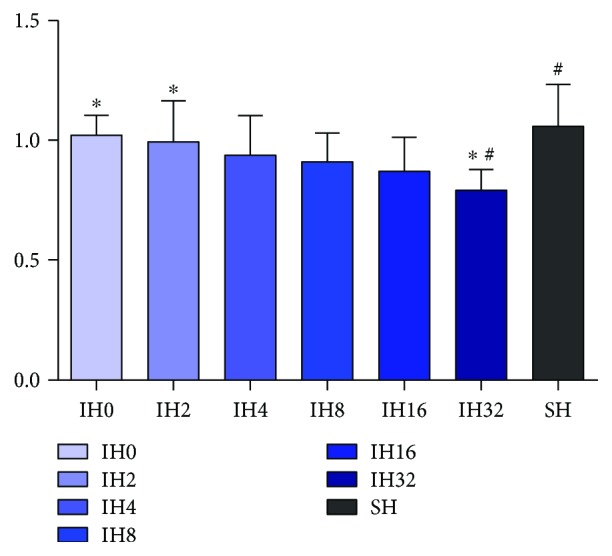
Effect of different numbers of IH cycles on IRS-1 mRNA expression (*n* = 9) (IH32 vs IH0 or IH2 ^∗^*P* < 0.05, IH32 vs SH ^#^*P* < 0.05).

In order to observe the effect of IH on insulin resistance of AGM-3T3-L1 adipocytes, qPCR was used to detect the expression level of IRS-1 mRNA. The results are shown in Figure 5. When AGM-3T3-L1 adipocytes were treated for IH32 cycles, the expression level of IRS-1 mRNA was decreased. Compared with the IH0 group and IH2 group, the difference was statistically significant (*P* < 0.05), and there was a significant difference compared with the SH group (*P* < 0.05). There was no significant difference among other groups (*P* > 0.05).

### 3.2. Effect of CIH on AGM-ApoE^−/−^ Mice

**Figure 6:**
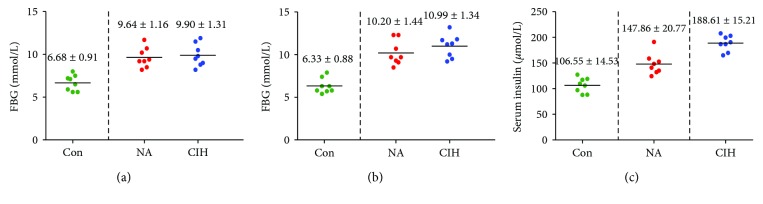
The level of FBG and serum insulin in the Con group, NC group, and CIH group. (a) FBG at CIH treatment at 4 w, (b) FBG at CIH treatment at 8 w, and (c) serum insulin at CIH treatment at 8 w.

For the general situation of experimental mice, the weight of mice was measured every 4 weeks. The start of feeding on high-fat diets was marked as D1. There was no statistical difference between the Con group, NC group, and CIH group on D1, high-fat diet induction at 4 w, CIH treatment at 4 w, and CIH treatment at 8 w. Secondly, we found that mice in the CIH group were observed to have a darker coat and less activity than were the other two groups during the experiment. Thirdly, the effect of CIH on blood glucose metabolism-related indicators, including FBG and serum insulin, was observed. FBG in the NC group and CIH group was significantly higher than that in the Con group at CIH treatment at 4 w and CIH treatment at 8 w (*P* < 0.05) (Figures 6(a) and 6(b)). The serum insulin levels in the NC group and CIH group were significantly higher than those in the Con group at CIH treatment at 8 w (*P* < 0.05), and that of the CIH group was higher than that of the NC group (*P* < 0.05) (Figure 6(c)).

**Figure 7:**
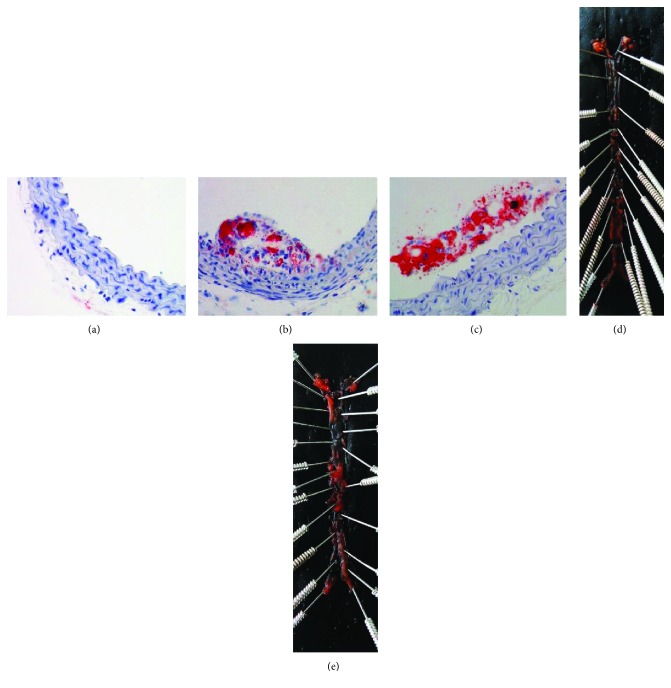
Aortic atherosclerotic plaques (Oil Red O staining). (a–c) Aortic atherosclerotic plaques in the ascending aorta: (a) Con group, (b) NC group, and (c) CIH group. (d, e) The longitudinal sections of the aorta in the NC and CIH group: (d) NC group and (e) CIH group.

In order to provide direct evidence for atherosclerosis, the Oil Red O staining method was used to contrast aortic atherosclerotic plaques in the ascending aorta of mice among each group. The following figures show the results of Oil Red O staining from the perspective of the blood vessel cross section; obvious AS plaques can be seen in the ascending aorta of the mice of the NC group and CIH group (Figures 7(a)–7(c)). We also compared Oil Red O staining in the longitudinal sections of the aorta in the NC and CIH groups to give a more intuitive version (Figures 7(d) and 7(e)). It can be seen that after CIH treatment, the number and area of aortic plaques in ApoE^−/−^ mice were significantly increased.

**Figure 8:**
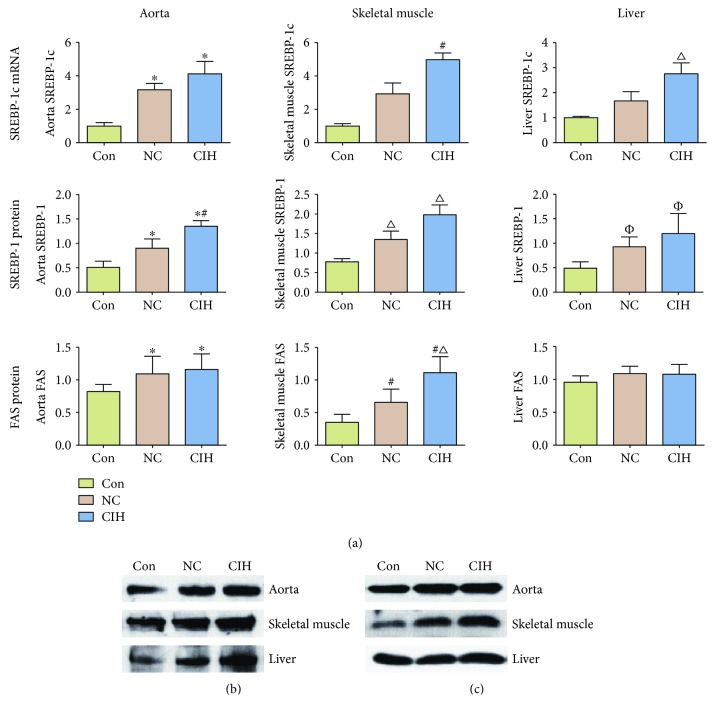
Effect of CIH treatment on SREBP-1c and FAS expression in different tissues (*n* = 6). (a) The first line showed SREBP-1c mRNA expression: aorta, compared with Con ^∗^*P* < 0.05; skeletal muscle, compared with Con ^#^*P* < 0.05; and liver, compared with Con ^*∆*^*P* < 0.05. The second line showed SREBP-1 protein expression: aorta, compared with Con ^∗^*P* < 0.05, compared with NC ^#^*P* < 0.05; skeletal muscle, compared with Con ^*∆*^*P* < 0.05; and liver, compared with Con ^*φ*^*P* < 0.05. The third line showed FAS protein expression: aorta, compared with Con ^∗^*P* < 0.05; skeletal muscle, compared with Con ^#^*P* < 0.05, compared with NC ^*∆*^*P* < 0.05; and liver, *P* > 0.05. (b) Representative Western blotting result of SREBP-1. (c) Representative Western blotting result of FAS.

To compare aortic SREBP-1c expression levels in different groups, qPCR and Western blotting were used to detect and compare mRNA and protein expression levels in the aorta, skeletal muscle, and liver in each group. The results of qPCR are shown in Figure 8(a). For the aorta, mRNA expression levels of SREBP-1c in NC and CIH groups were statistically higher than those in the Con group (*P* < 0.05). For the skeletal muscle and liver, the mRNA expression level of SREBP-1c in the CIH group was significantly higher than that in the Con group (*P* < 0.05). The results of Western blotting are shown in Figures 8(a) and 8(b). For the aorta, the protein expression levels of SREBP-1 in NC and CIH groups were statistically higher than those in the Con group (*P* < 0.05), and the CIH group was statistically higher than the NC group as well (*P* < 0.05). However, there was no statistically significant difference between the CIH group and the NC group in the protein expression levels of SREBP-1 of the skeletal muscle and liver (*P* > 0.05).

Then, the protein expression level of FAS which is a downstream molecule of SREBP-1c was detected as well. The results are shown in Figures 8(a) and 8(c). For the aorta and skeletal muscle, the protein expression levels of FAS in the NC and CIH groups were statistically higher than those in the Con group (*P* < 0.05). And the CIH group was statistically higher than the NC group in skeletal muscle (*P* < 0.05). In the liver, there was no statistical difference in FAS protein expression between these groups (*P* > 0.05).

**Figure 9:**
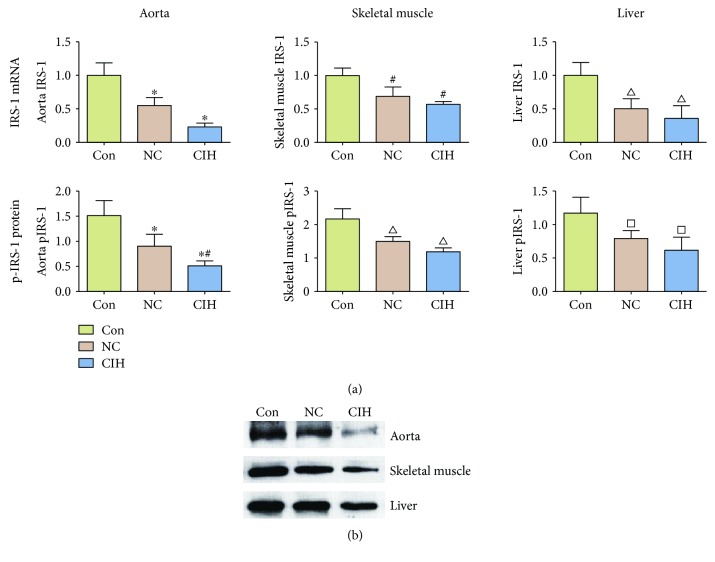
Effect of CIH treatment on IRS-1 mRNA expression and pIRS-1 expression in different tissues (*n* = 6). (a) The first line showed IRS-1 mRNA expression: aorta, compared with Con ^∗^*P* < 0.05; skeletal muscle, compared with Con ^#^*P* < 0.05; and liver, compared with Con ^*∆*^*P* < 0.05. The second line showed pIRS-1 protein: aorta, compared with Con ^∗^*P* < 0.05, compared with NC ^#^*P* < 0.05; skeletal muscle, compared with Con ^*∆*^*P* < 0.05; and liver, compared with Con ^□^*P* < 0.05. (b) Representative Western blotting result.

The effects of CIH on IRS-1 mRNA and p-IRS-1 protein expression levels in the aorta, liver, and skeletal muscles in AGM-ApoE^−/−^ mice are shown in Figure 9(a). The mRNA expression levels of IRS-1 in the aorta, skeletal muscle, and liver were significantly lower in the NC and CIH groups than those in the Con group (*P* < 0.05), while there was no statistically significant difference between the CIH and NC groups (*P* > 0.05). The results of Western blotting are shown in Figures 9(a) and 9(b). For the aorta, the protein expression levels of p-IRS-1 in the NC and CIH groups were statistically higher than those in the Con group (*P* < 0.05), and the CIH group was statistically higher than the NC group as well (*P* < 0.05). However, there was no statistically significant difference between the CIH group and the NC group in the protein levels of p-IRS-1 of the skeletal muscle and liver.

## 4. Discussion and Conclusion

OSAHS can cause comprehensive damage to various systems of the body, among which secondary cardiovascular disease is the most common and severe. OSAHS is an independent risk factor for cardiovascular disease and could lead to increased occurrence of hypertension, stroke, myocardial infarction, heart failure, arrhythmias, and sudden death in the night, and these diseases are the main reason that OSAHS eventually causes disability or death [4–6]. CIH is the core pathological basis of OSAHS, while CIH and insulin resistance-based disorders of glucose and lipid metabolism disorder often occur in patients of OSAHS at the same time. CIH and abnormal glucose metabolism (AGM) cooperate to promote the injury of the vascular endothelium and secondary atherosclerosis (AS) in OSAHS and then promote the development of OSAHS-related cardiovascular and cerebrovascular diseases [7–10]. Based on the situation that CIH and AGM often coexist in patients with OSAHS and jointly lead to AS, we intend to establish a composite model of CIH and AGM in this study to help explain the effect of CIH on AGM and their synergistic effect on AS injury.

In the animal research part of this study, the hypoxic animal incubator (YCP-160D) was used to treat AGM-ApoE^−/−^ mice with a regular pattern of gas circulation to simulate CIH, of which the experimental operation was relatively simple and controllable. The CIH stimulation took 180 s as one cycle, which means about 20 hypoxic episodes per hour, which is roughly equivalent to human moderate OSAHS [11]. We observed that CIH composite AGB has a significant effect on promoting AS progression and is significantly stronger than that caused by simple AGB factors. Thus, we established a relatively reliable CIH + AGB-ApoE^−/−^ mouse model. On the other hand, we also observed that CIH could upregulate the serum insulin level in AGM-ApoE^−/−^ mice and inhibit the expression and phosphorylation of IRS-1, showing the effect of promoting insulin resistance, which may also be an important factor leading to the progression of AS.

In recent years, the role of sterol regulatory element-binding proteins (SREBPs) in the development of AS has been gaining attention [12, 13]. SREBPs play an important role in cholesterol anabolism and regulation of cholesterol balance, being considered as key molecules in relation to abnormal glucose and lipid metabolism [14]. SREBPs are a type of “basic helix-loop-helix-leucine zipper” (bHLH-ZIP) protein that specifically binds to sterol regulatory element (SRE-1). SREBPs include SREBP-1 and SREBP-2, while the former includes SREBP-1a and SREBP-1c isoforms, which are mainly expressed in the liver and adipocytes, mainly involved in the regulation of cholesterol and fatty acid biosynthesis. Among them, SREBP-1c is widely expressed in the liver, adipose tissue, skeletal muscle, adrenal glands, etc., playing a more important role and having more abundant research evidence in current studies [15]. The SREBPs are anchored on the endoplasmic reticulum membrane and combined with the SREBP cleavage-activating protein (SCAP) to form the SCAP/SREBP complex. Intracellular hypocholesterol, insulin, insulin-like growth factor I, etc., can promote the transfer of SCAP/SREBP complexes to the Golgi apparatus to become nuclear SREBP (nSREBP). The latter rapidly migrates into the nucleus and binds to SRE-1, activating its target genes such as the low-density lipoprotein receptor (LDLR) gene, the acetyl-CoA carboxylase (ACC) gene, the fatty acid synthase (FAS) gene, and transcription and expression of glucokinase (GK) genes. In our study, we observed the effects of different degrees of IH stimulation on SREBP-1-related signaling molecules mainly through the cell experiments *in vitro*.

There, what role does SREBP-1 play in IH impacting on AGM and even AS? In the first place, there is a correlation between IH and SREBPs. Current studies suggest that IH can activate SREBPs through complex molecular pathways. Oxidative stress and inflammatory response are often observed as prominent effects caused by IH. On the one hand, the levels of ROS and HIF-1*α* increased significantly under IH conditions [16, 17]. IH may upregulate the HIF-1*α*/SREBP-1c/FAS pathway through the ROS pathway, leading to abnormal lipid metabolism, while SCAP has a linker site for HIF-1, and HIF-1 may also regulate SREBP expression through this pathway. On the other hand, IH-mediated low-level inflammation in various tissues of the body is also associated with SREBPs. IH can cause an upregulation of NF-*κ*B signaling pathways [18], while other studies suggest that NF-*κ*B expression levels and SREBP expression have a positive correlation [19]. In our study, IH enhanced the expression level of SREBP-1c and its downstream FAS mRNA and protein in AGM-3T3-L1 adipocytes, especially in the IH cycle more than 8 times. There was a positive correlation between the upregulation of SREBP-1c levels and the increase in the number of IH cycles, and the effect was greater than that of SH at the same stimulation time. In the second place, SREBPs play a pivotal role in insulin resistance and abnormal glucose and lipid metabolism. Studies have shown that upregulation of SREBP-1c may restrain the expression of IRS and its downstream signaling molecule Akt and suppress insulin sensitivity, while drugs that exert anti-inflammatory effects through the SREBP-1c pathway can significantly improve pancreatic *β*-cell function and produce antidiabetic effects [20]. In umbilical vein endothelial cells (HUVEC), inhibiting the expression of SREBP-1c and its downstream pathways including the FAS gene can reduce the vascular proinflammatory responses associated with metabolic abnormalities, thereby inhibiting the occurrence of atherosclerosis [12]. In addition, the main function of SREBP-1c is to modulate fatty acid metabolism, which is mainly achieved through the regulation of fat metabolism-related genes such as FAS. In summary, the current study can prove that SREBP-1c is closely related to insulin resistance and abnormal metabolism of glucose and lipids, while this correlation has not been discussed under the condition of IH. Our research is based on this and found that IH promotes the expression of SREBP-1c in adipocytes while inhibiting the expression of IRS-1 and inhibiting the uptake and utilization of glucose. At the level of animals *in vivo*, as well, CIH stimulation can upregulate the expression of SREBP-1c and FAS in the aorta, skeletal muscle, and liver.

As we described above for the role SREBP-1 molecules play in this process, our study examined the effect of CIH on the expression of SREBP-1 signaling molecules in the aorta, skeletal muscle, and liver. This is the first time that this effect of CIH has been observed at the level of animal experiments and has not been reported in the current study. In our study, the aorta was used as the main observation object. It was observed that CIH composite AGM promoted the expression of SREBP-1c and FAS in the aorta, inhibited the expression and phosphorylation of IRS-1, and had an obvious AS-promoting effect. Therefore, the effect of AS promotion might be related to the promotion of the expression of SREBP-1c-related signaling molecules in arterial vessels. In addition, we also observed that CIH composite AGM significantly promoted the expression of the SREBP-1c/FAS signaling pathway in the skeletal muscle and observed its inhibition of IRS-1 expression and phosphorylation at the same time. Previous studies have investigated the upregulation of SREBP-1c/FAS expression and downregulation of p-IRS-1 expression in cultured insulin resistance muscle cells *in vitro*, and drugs such as metformin, which inhibit the expression of SREBP-1c and FAS, can improve insulin resistance [21]. Then, CIH-mediated studies of the relationship between IR and SREBP-1c/FAS signaling pathways are also of further concern. Therefore, studies on the relationship between CIH and SREBP-1-related signaling pathways are also worth more attention in improving skeletal muscle insulin resistance. Finally, the effect of CIH composite AGM on the expression of the hepatic SREBP-1-related signaling pathway and the effect of inhibiting hepatic IRS-1 expression and phosphorylation levels were also observed. Thus, it was speculated that CIH composite AGM may promote insulin resistance and lipid deposition of the liver through the SREBP-1-related signaling pathway. This may provide some new ideas for the current study of SREBP-1c and nonalcoholic fatty liver disease (NAFLD), obesity, and OSAHS [22, 23].

In summary, an animal model of a CIH composite AGM-mediated AS animal model was established in our study. And we found that CIH composite AGM could promote the progress of AS, which might be related to the modulation of the expression of SREBP-1-related molecular pathways.

## Data Availability

The data used to support the findings of this study are included within the article.
